# Peritoneal Volume Influence on POCUS Volume Assessment in Patients Undergoing Peritoneal Dialysis

**DOI:** 10.24908/pocusj.v10i02.19345

**Published:** 2025-11-17

**Authors:** María Muñiz Rincón, Diego Barbieri, Virginia López de la Manzanara, Elena Ruiz Ferreras, Arianne Aiffil Meneses, Carlos Fernández Fernández, Cristina Riaza Ortiz, Rómulo Loayza López, Jose Antonio Herrero Calvo, Ana I. Sanchez-Fructuoso

**Affiliations:** 1 Department of Nephrology, Hospital Universitario Clínico San Carlos, Madrid, Spain.

**Keywords:** Overhydration, Venous excess ultrasonography, Point of care ultrasonography, Peritoneal dialysis, Lung ultrasonography

## Abstract

**Objective:**

This study aimed to investigate variations in fluid overload using point of care ultrasound (POCUS). We assessed patients undergoing peritoneal dialysis (PD) with full and drained peritoneum and their correlation with clinical parameters.

**Methods:**

POCUS examination and intra-abdominal pressure (IAP) measurements were conducted in patients undergoing PD with a full peritoneum. Subsequently, after drainage, a new POCUS and bioimpedance analysis (BIA) were performed.

**Results:**

Seventeen patients were included in the study: 70.6% male, mean age 66+/-9.5 years. Of these, 65% had fluid overload >1 L and 23% had overhydration (OH) adjusted for extracellular water (ECW) exceeding 15%, as assessed by BIA. B-lines with full peritoneum had a median of 1 (0-2.5), and with drained peritoneum 0 (0-0). This difference was not statistically significant (p=0.063). In the correlation analysis of variables, IAP and PD fluid volume per m2 of body surface did not correlate with the diameters or collapsibility of the inferior vena cava (IVC) with full or drained peritoneum. The degree of OH in liters correlated with IVC collapsibility with drained peritoneum (Spearman ρ=-0.43; p=0.08), as did the OH adjusted for ECW (Spearman ρ=-0.61; p=0.02). These correlations disappeared with full peritoneum (p>0.05).

**Conclusions:**

There were no significant differences in ultrasound volume overload parameters in patients undergoing PD with full vs. drained peritoneum. However, there are indications of lower POCUS sensitivity to fluid overload with a full peritoneum.

## Introduction

Point of care ultrasound (POCUS) is increasingly recognized as a valuable bedside diagnostic tool that enhances physical examination and facilitates clinical decision-making [1]. One of the most common applications of POCUS is volume status assessment. It is particularly useful as an adjunct to physical examination, given the limited sensitivity of traditional clinical signs such as dyspnea, orthopnea, jugular vein distention, edema, or crackles [1]. A meta-analysis evaluating the diagnostic accuracy of acute heart failure in emergency departments reported low sensitivity for classic symptoms like orthopnea (52%) and for radiographic signs such as pulmonary edema on chest X-ray (56.9%) [2]. Furthermore, despite the markedly elevated pulmonary capillary wedge pressures observed in patients with heart failure, radiographic evidence of pulmonary congestion is absent in 39% of cases [3].

Volume status estimation is particularly relevant in nephrology, as volume overload is a frequent complication in patients with end-stage kidney disease and those undergoing dialysis. It is associated with hypertension, increased arterial stiffness, left ventricular hypertrophy, impaired oxygenation, and heightened cardiovascular morbidity and mortality [4].

In patients on peritoneal dialysis (PD), volume assessment presents an additional challenge due to the prognostic significance of preserving residual diuresis [5–7]. Consequently, accurate evaluation is crucial for maintaining optimal volume status, facilitating the preservation of residual renal function while preventing overhydration (OH).

A key difficulty in congestion assessment lies in differentiating between vascular and tissue congestion, which is essential for guiding therapeutic interventions [8]. POCUS has emerged as a useful tool for evaluating both intravascular and extravascular fluid overload through three principal strategies: lung POCUS, which enables rapid and accurate assessment of tissue congestion; the Venous Excess Ultrasound (VExUS) grading system, which utilizes venous Doppler imaging to grade vascular congestion; and cardiac POCUS, which assesses cardiac and valvular morphology and function [9].

However, certain limitations of POCUS in volume assessment must be acknowledged [10]. In patients with elevated intra-abdominal pressure (IAP), a collapsed inferior vena cava (IVC) may be observed despite high right atrial pressure, potentially leading to an underestimation of fluid overload [11]. This limitation is particularly relevant in patients on PD, where evidence on the use of POCUS for hydration assessment remains scarce. Additionally, the presence of peritoneal dialysate, which mimics “pseudo-ascites,” could interfere with ultrasound-based measurements and IVC diameter assessment. Given these potential challenges, this study aimed to determine whether the PD technique introduces artifacts or measurement inconsistencies. Ultimately, we hoped to refine the interpretation of POCUS findings in clinical practice.

## Methods

This single-center observational study aimed to evaluate differences in POCUS-based volume overload parameters in patients on PD, comparing measurements taken with a full peritoneum vs. after drainage.

All patients from our PD unit at Hospital Clínico San Carlos were screened for inclusion. The inclusion criteria were age >18 years and dialysis vintage on PD >3 months. Patients were excluded if they had experienced an acute illness in the three months prior to screening (e.g., peritonitis, hospitalization for any cause, or catheter malfunction).

Of the 32 patients undergoing PD at our hospital, 17 met the inclusion criteria and were enrolled in the study. Approval from our local Ethics Committee was granted. Informed consent was obtained from all participants prior to their inclusion in the study.

**Figure 1.**
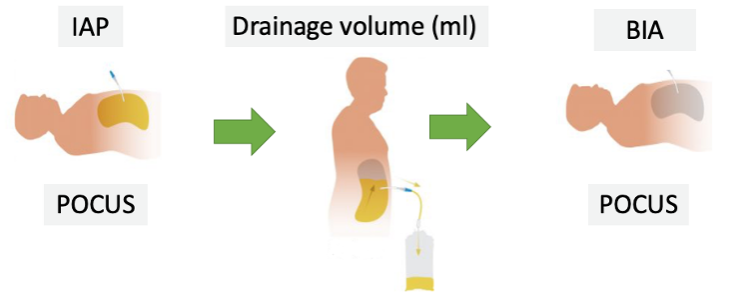
Experimental protocol

Each patient was scheduled for evaluation in the morning with the night dwell still in place. Initially, volume overload was assessed using POCUS (protocol detailed below). Following this, IAP was measured, and the PD fluid was drained. After drainage, a second POCUS examination and a bioimpedance analysis (BIA) were performed. A schematic representation of the study protocol is provided in Figure 1.

IAP was measured by trained nurses using the non-invasive method described by Durand in 1992 [12]. This technique involves a simple, bedside hydrostatic method. The measurement is performed with the patient in the supine position before drainage of the dialysate. The external end of the PD catheter is connected to a sterile extension set, which is then held vertically. The height of the dialysate column in the tubing, relative to a reference point at the midaxillary line (usually at the level of the iliac crest), is measured in centimeters of water (cm H_2_O). This value represents the intraperitoneal pressure.

This method is considered safe, reproducible, and sufficiently accurate for clinical use in PD, as validated by subsequent studies comparing it to direct pressure measurements. The normal value for intraperitoneal pressure with a 2 L fill is approximately 12 ± 2 cm H_2_O, with linear increases for larger volumes. The technique is widely used in clinical practice to assess tolerance to intraperitoneal volume and to guide prescription adjustments in patients on PD [13].

Body composition and hydration status were assessed using a portable whole-body bioimpedance spectroscopy device (BCM—Fresenius Medical Care D, Schweinfurt, Bavaria, Germany), which measures impedance at 50 different frequencies. This method detects fluid overload by measuring the body's resistance and reactance to a small, painless electrical current. These measurements allow estimation of total body water, extracellular water (ECW), intracellular water and total or relative OH [14]. Measurements were taken after drainage, following 15 minutes of rest in the supine position. Two criteria were used to define OH: OH volume >1 liter (OH>1L) or OH volume >15% of ECW volume (OH/ECW>15%) [15].

Tissue congestion was estimated using lung POCUS, and vascular congestion was estimated using VExUS. Cardiac POCUS was not performed due to lack of operator experience.

### Lung POCUS protocol

B-lines were identified using B-mode ultrasound with a convex probe (SonoSite Edge II - FUJIFILM SonoSite, Inc., Bothell, Washington, USA) applied longitudinally (including ribs in the scanning field) while the patient was in a seated position. A 16-zone protocol was employed, dividing each hemithorax into 8 regions: 4 in the frontal and 4 in the dorsal planes. In the frontal regions, measurements were taken anteriorly and laterally following the simplified 8-lung field model proposed by Torino et al. [16]. In the dorsal regions, measurements were obtained at the apex (medial and lateral) and lung bases (medial and lateral) to enhance diagnostic sensitivity. We counted the total (absolute) number of B lines and the number of positive B-line fields (≥3/field).

### VExUS protocol

IVC diameter and collapsibility were assessed using B-mode ultrasound, along with spectral Doppler measurements of the hepatic vein and portal veins. Renal Doppler measurements as part of the VExUS protocol were excluded due to concerns regarding its reliability in advanced chronic disease, where parenchymal and vascular stiffness may affect accuracy [10]. Congestion severity was classified into four grades according to the VExUS protocol: no congestion (grade 0), mild congestion (grade 1), moderate congestion (grade 2), and severe congestion (grade 3) [17].

Although hepatic vein and portal vein patients are indicated only when the IVC is >2 cm, we performed hepatic vein and portal vein Doppler in all patients.

*Other baseline data collected*

- *Demographics*: age, sex, weight, height, and body mass index (BMI).
- *Clinical history*: comorbidities, with emphasis on congestive heart failure (CHF), chronic obstructive pulmonary disease (COPD), and chronic liver disease (CLD).
- *Heart failure classification*: functional status was obtained using the New York Heart Association (NYHA) classification.
- *Dialysis details*: PD vintage, last recorded peritoneal equilibration test (PET) and PD prescription.

### Statistical analysis

Continuous variables were presented as median and interquartile range (IQR), while categorical variables were expressed as counts and percentages. Given the small sample size (n<30), non-parametric tests were used.

Differences in ultrasound parameters between the full and empty peritoneum states were analyzed using the Wilcoxon signed-rank test. Subgroup analyses were performed to compare patients with OH>1L or OH/ECW>15% by BIA vs. OH<1L or OH/ECW<15%, respectively. Correlations between ultrasound parameters and clinical variables were assessed using Spearman's Rho.

All statistical analyses were performed using SPSS, version 21.0 (IBM Corp., Armonk, NY, USA).

## Results

### Patient Characteristics

A total of 17 out of 32 patients in our PD unit were included in the study. Baseline characteristics of these patients are shown in Table 1. The median age was 69 years (5 IQR 59.5-75), and the majority (70.6%) of cases were male. The median BMI was 27 (IQR 24,4-32,3). Only 1 patient (5.8%) was on automated PD, and 16 patients (94.2%) were on continuous ambulatory PD. The median dialysis vintage on PD was 22 months (IQR 14-40). The majority of patients (82.4%) had icodextrin prescribed. Median results were 0.73 (IQR 0.705-0.785).

**Table 1.** Baseline characteristics. BMI: Body Max Index; PD: Peritoneal dialysis; CAPD: Continuous ambulatory peritoneal dialysis; LVEF: Left ventricular ejection fraction; NYHA: New York Heart Association, PASP: Pulmonary artery systolic pressure; OH>1L: Overhydration exceeding 1 liter; OH/EWC>15%: Overhydration adjusted for extracellular water (ECW) exceeding 15%; BIA: Bioelectrical impedance analysis; IAP: Intra-abdominal pressure.

Characteristics	Value
Sex (Male)	70.6%
Age (years) Median (IQR)	69 (59.5–75)
BMI Median (IQR)	27 (24.4–32.28)
Time in PD (months) Median (IQR)	22 (14–40)
CAPD	76.5%
Icodextrin	82.35%
Chronic Heart Failure	35.3%
LVEF (%) Median (IQR)	60 (58–64)
Functional class (NYHA classification) Median (IQR)	2 (1–2)
PASP (mmHg) Median (IQR)	20 (20–24.5)
OH >1 L by BIA	65%
OH/ECW>15% by BIA	23.5%
OH (L) Median (IQR)	1.7 (0.1–2.45)
OH/ECW (%) Median (IQR)	10.2 (1.25–15.05)
Chronic Obstructive Pulmonary Disease	5.9%
Cirrhosis	0%
IAP (mmHg) Median (IQR)	13.5 (12.5–18.5)

Six out of seventeen patients (35.5%) had diagnosed CHF, with a median NYHA functional class of 2 (1-2). The median left ventricular ejection fraction (LVEF) was 60% (IQR 58-64) and the median pulmonary artery systolic pressure (PASP) was 20 mmHg (IQR 20-24.5). Eleven out of seventeen (65%) had fluid overload >1L and four out of seventeen (23%) had OH adjusted for ECW exceeding 15%, as assessed by BIA. The median IAP was 13.5 mmHg (IQR 12.5-18.5).

### POCUS Results

The descriptive results of the POCUS parameters are summarized in Table 2. The median maximum diameter of the IVC with full and drained peritoneum was 1.47 cm (IQR 1.09-1.70) and 1.57 cm (IQR 1.22-1.85) respectively, with no significant difference (p=0.179). The percentage of collapsibility of IVC with a full and drained peritoneum was 54.7% (IQR 29.17-67.42) and 48% (IQR 35.7-55.78) respectively, also showing no significant difference (p=0.59). The pattern of congestion in the hepatic vein was graded as a median of 1 (IQR 1-1) for both full and drained peritoneum (p=0.56), and the portal vein also showed a median of 1 (IQR 1-1) for both conditions (p=0.32).

**Table 2.** Full peritoneum VS after drainage: Median (IQR). IVC: Inferior vena cava.

Variable	Full	After drainage	p
Maximum diameter IVC (cm)	1.47 (1.09–1.70)	1.57 (1.22–1.85)	0.18
Minimum diameter IVC (cm)	0.86 (0.32–1.10)	0,77 (0.56–0.99)	0.76
IVC collapsibility (%)	54.7 (29.17–67.42)	48 (35.7–55.78)	0.59
Hepatic vein	1 (1–1)	1 (1–1)	0.56
Portal vein	1 (1–1)	1 (1–1)	0.32
B-lines (n)	1 (0–2.50)	0 (0–0)	0.063

Regarding B-lines, the median number of total (absolute) B-lines was 1 (IQR 0-2.5) with a full peritoneum, while it was 0 (0-0) with a drained peritoneum (p=0.063). The disappearing B-lines after drainage were all originally located in the medial basal lung fields. No differences were detected in the number of positive B-line fields between full and drained peritoneum (p>0.05)

We considered performing additional subgroup analyses based on volume overload as assessed by BIA (OH>1L or OH/ECW>15%). However, this approach was ultimately dismissed due to the limited sample size.

### Correlation Analysis

In the correlation analysis (Tables 3 and 4), IAP and PD fluid volume per m^2^ of body surface did not show a significant correlation with IVC diameter or collapsibility with either full or drained peritoneum. OH in liters (OH (L)) by BIA tended to correlate with IVC collapsibility with a drained peritoneum (Spearman ρ=-0.43; p=0.08), though this did not reach statistical significance. However, OH/ECW by BIA significantly correlated with IVC collapsibility with a drained peritoneum (Spearman ρ =-0.61; p=0.02). These correlations were not observed with a full peritoneum (p>0.05).

**Table 3.** Correlations with IVC collapse in full peritoneum and after drainage (ρS: Spearman's Rho). IVC: Inferior vena cava; BSA: Body surface area; IAP: Intra-abdominal pressure; OH: Overhydration; BIA: Bioelectrical impedance analysis; OH/ECW: Overhydration/Extracellular water.

Variable	% Collapse IVC full peritoneum		% Collapse IVC after drainage	
	ρS	p	ρS	p
Dialysate volume (ml/m2 BSA)	−0.025	0.92	−0.01	0.51
IAP (mmHg)	−0.254	0.32	−0.28	0.27
OH (L) by BIA (after drainage)	−0.009	0.97	−0.43	0.08
OH/ECW by BIA (%) (after drainage)	0.103	0.72	−0.61	0.02
Differential of maximum diameter IVC (cm)	−0.6	0.01	−0.13	0.60
Differential of minimum diameter IVC (cm)	−0.703	0.002	0.1	0.69

**Table 4.** Correlations with the maximum and minimum diameter of IVC (ρS: Spearman's Rho). IVC: Inferior vena cava; BSA: Body surface area; IAP: Intra-abdominal pressure; OH: Overhydration; BIA: Bioelectrical impedance analysis; OH/ECW: Overhydration/Extracellular water.

Variable	Differential of maximum diameter IVC (cm)		Differential of minimum diameter IVC (cm)	
	ρS	p	ρS	p
Dialysate volume (ml/m^2^ BSA)	0.221	0.39	0.96	0.71
IAP (mmHg)	0.236	0.36	0.224	0.38
OH (L) by BIA (after drainage)	0.028	0.91	−0.245	0.34
OH/ECW by BIA (%) (after drainage)	0.09	0.76	−0.275	0.34
Collapse IVC full peritoneum (%)	−0.6	0.01	−0.703	0.002*
Collapse IVC after drainage (%)	−0.135	0.6	0.1	0.69

Again, we considered performing additional subgroup analyses based on volume overload as assessed by BIA (OH>1L or OH/ECW>15%), but this approach was dismissed due to the limited sample size.

## Discussion

POCUS has become increasingly useful for assessing volume overload over the past three decades. Initially, lung POCUS outperformed chest radiographs and N-terminal pro B-type natriuretic peptide in diagnosing acute heart failure, with B-lines at discharge linked to higher readmission and mortality [18,19]. In chronic heart failure, lung POCUS-guided diuretic titration reduced hospitalizations, though mortality benefits are unclear [20,21]. Since 2020, the VExUS protocol has standardized venous congestion assessment, correlating with renal recovery in acute kidney injury [22].

In hemodialysis (HD), POCUS is valuable in assessing fluid overload, guiding ultrafiltration, and adjusting dry weight [23–27]. Repeated IVC assessment helps differentiate hypotension due to low effective circulating volume caused by autonomic dysfunction or peripheral vasodilation [23–25]. The LUST (Lung water by Ultra-Sound guided Treatment) trial investigated whether lung ultrasound–guided fluid management could improve outcomes in patients with end-stage renal disease at high cardiovascular risk. Although the primary outcomes remained unchanged, lung POCUS–guided ultrafiltration was associated with fewer heart failure episodes, less intradialytic hypotension, and improved left ventricular function [26,27]. However, its application in PD has received limited attention, due to differences in fluid elimination dynamics and body fluid compartmentalization [28].

Granata et al. summarized key POCUS applications in PD, including the diagnosis of exit-site infections, catheter-related complications, and technique-related issues such as abdominal hernias, pleural defects, and peritonitis [29]. Koratala et al. later introduced its use for volume assessment but did not highlight PD-specific considerations [30]. Given the scarce literature, our study aimed to evaluate the impact of PD fluid and increased IAP on POCUS findings.

Systematic lung POCUS use has demonstrated benefits in HD patients [28]. Thus, we focused on differences in B-lines between full and drained peritoneum states. Our results showed a B-line count with a full peritoneum. While this difference did not reach statistical significance (p=0.063), it suggests a potential association. A bigger sample size may have revealed statistical significance.

Notably, the B-lines with a full peritoneum that disappeared after drainage were all located in the medial basal lung fields. If this result is repeatedly proven in future studies it may be hypothesized that those B-lines are related to the intraperitoneal fluid, due to passive basal atelectasis from increased IAP. If confirmed in future studies, this finding suggests that some basal lung B-lines may be expected in patients on PD, potentially dissociating them from fluid overload in this specific scenario. This would imply that reducing weight or increasing ultrafiltration based solely on these findings may not always be necessary.

A previous study indicated that lung POCUS outperformed conventional physical examination and BIA in detecting fluid overload in patients on PD [31]. They also reported a strong association between lung congestion, ejection fraction, and left atrial volume. It is important to note that a key limitation of BIA is its inability to detect the location of extracellular volume expansion (e.g., pulmonary edema vs. ascites vs. venous congestion) [32]. Differing from this, lung POCUS provides a direct assessment of lung water content, which correlates with pulmonary wedge pressure, and is a crucial determinant of left ventricular filling pressure [32]. Our results do not necessarily contradict these findings, as B-line count is still expected to correlate with OH. Guided ultrafiltration by lung POCUS in patients on PD can still be useful. If our results are confirmed in future studies, it will be important to consider whether lung POCUS is performed with a full or empty peritoneum.

Elevated IAP has been shown to affect IVC size independent of volume status [33]. This can compress and deform the IVC and reduce inspiratory variation [34]. In cirrhosis and portal hypertension, Doppler evaluation of the portal vein primarily reflects local pressure changes rather than right atrial pressure, potentially leading to misinterpretation [35,36]. A similar “pseudo-cirrhosis” phenomenon may occur in PD when the peritoneum is full (although with much lower intraperitoneal volumes). In our study, paired data analysis did not reveal statistically significant differences in the maximum diameter differential between a full and drained peritoneum, although there was a quantitative median difference of 0.1 cm (p=0.18). Likewise, IVC collapsibility was 7-8% higher when the peritoneum was full, though this was not statistically significant.

Our findings highlight a discrepancy between the high prevalence of fluid overload detected by BIA (65% with OH>1L) and the lack of echographic congestion markers (median values indicating no or mild congestion). While many patients exhibited OH>1L, few demonstrated significant overload adjusted for their ECW (only 4 patients (23%) had OH/EWC>15%). Furthermore, as observed in the results (Table 2), there were no extreme or clinically evident degrees of fluid overload. These findings could explain the lack of echographic evidence of fluid overload. This distinction between OH>1L and OH/ECW>15% is significant, as OH/ECW>15% has been shown to be a risk factor for mortality and morbidity in patients undergoing HD [37].

When analyzing the relationship between different variables in our study, we were surprised to find that results differed from our expectations. In particular, the patterns observed in other patients with increased IAP, such as those with cirrhosis, varied. Initially, we investigated how specific factors might influence changes in the diameter of the IVC when the peritoneum was drained (Table 3). Surprisingly, we found that IAP and the volume of dialysis adjusted for body surface area did not show any significant correlation with changes in IVC diameter from full to drained peritoneum. In fact, we did not find any clinical variables, including fluid overload assessed by BIA, that were associated with changes in IVC diameter from full to drained peritoneum.

The second aspect we analyzed was the correlation between the collapse of the IVC when emptying the peritoneum and various clinical variables (Table 4). Again, we did not find any correlation with IAP and the volume of dialysis adjusted for body surface area. This discrepancy between our findings and what we would expect is likely related to measurement challenges in the presence of free intra-abdominal fluid (hepatic fluid), rather than necessarily being linked to increased pressure or volume within the abdomen. However, other arguments may explain the absence of changes from full to drained peritoneum. Since the median IAP in our study was within a normal range for patients undergoing PD (13.5 cm H_2_O), it may not have reached the critical status to produce changes in POCUS measurements. This would imply that PD dialysate would impact abdominal POCUS if IAP is within normal range in patients on PD. Another consideration is that repeated measurements after longer waiting times (e.g., 1-hour after drainage) may show changing results due to solute redistribution (between the interstitium and intravascular compartments, for example). This should be considered for future studies.

Notably, the correlation between IVC collapsibility and OH was only observed when the peritoneum was drained, but it lost significance when the peritoneum was full. This suggests that IVC collapsibility may not be a reliable marker of OH in patients undergoing PD with a full peritoneum. Clinically, this implies that peritoneal drainage before POCUS assessment may be preferable, though confirmatory studies are needed.

Renal vein Doppler, a component of the VExUS score, has been shown to correlate with right atrial pressure and poor outcomes in congestive patients [38]. However, we excluded this measurement due to concerns regarding its reliability in advanced chronic disease, where parenchymal and vascular stiffness may affect accuracy [10]. Internal jugular vein echographic parameters (e.g., collapsibility or maximum diameter) were not performed in our study since they are not part of the VExUS score, but have proven to be valuable and easy-to-perform for fluid overload and high central venous pressure [39]. Future studies may consider including internal jugular vein POCUS.

Our study has limitations. The small sample size limits the generalizability of our findings. Given the exclusion criteria, our results may only apply to stable ambulatory patients on PD and not to acute or decompensated patients. Additionally, POCUS was performed by two nephrologists with limited training rather than by radiologists or expert sonographers. However, in clinical practice, POCUS is often conducted by non-expert clinicians with minimal formal training. Finally, some of the conclusions are driven by the correlations results that were performed in a high number, which creates a risk of type I error (or false positives).

## Conclusions

Our study suggests that POCUS may have reduced reliability in detecting fluid overload in patients undergoing PD with a full peritoneum. While our findings indicate a potential impact of IAP on ultrasound assessments, further clinical trials are necessary to confirm these observations and refine POCUS applications in PD.
